# Identification and characterization of *Toxoplasma gondii* aspartic protease 1 as a novel vaccine candidate against toxoplasmosis

**DOI:** 10.1186/1756-3305-6-175

**Published:** 2013-06-14

**Authors:** Guanghui Zhao, Aihua Zhou, Gang Lu, Min Meng, Min Sun, Yang Bai, Yali Han, Lin Wang, Huaiyu Zhou, Hua Cong, Qunli Zhao, Xing-Quan Zhu, Shenyi He

**Affiliations:** 1 Department of Parasitology, Shandong University School of Medicine, Jinan, Shandong Province, 250012, People’s Republic of China; 2 Department of Pediatrics, Provincial Hospital Affiliated to Shandong University, Shandong University School of Medicine, Jinan, Shandong Province, 250012, People’s Republic of China; 3 State Key Laboratory of Veterinary Etiological Biology, Key Laboratory of Veterinary Parasitology of Gansu Province, Lanzhou Veterinary Research Institute, CAAS, Lanzhou, Gansu Province, People’s Republic of China

**Keywords:** Toxoplasma gondii, Aspartic protease, Bioinformatics, Vaccine, Toxoplasmosis

## Abstract

**Background:**

*Toxoplasma gondii* is an obligate intracellular parasite that can pose a serious threat to human health by causing toxoplasmosis. There are no drugs that target the chronic cyst stage of this infection; therefore, development of an effective vaccine would be an important advance. Aspartic proteases play essential roles in the *T. gondii* lifecycle. The parasite has four aspartic protease encoding genes, which are called toxomepsin 1, 2, 3 and 5 (TgASP1, 2, 3 and 5, respectively).

**Methods:**

Bioinformatics approaches have enabled us to identify several promising linear-B cell epitopes and potential Th-cell epitopes on TgASP1, thus supporting its potential as a DNA vaccine against toxoplasmosis. We expressed TgASP1 in *Escherichia coli* and used the purified protein to immunize BALB/c mice. The antibodies obtained were used to determine where TgASP1 was localized in the parasite. We also made a TgASP1 DNA vaccine construct and evaluated it for the level of protection conferred to mice against infection with the virulent RH strain of *T. gondii*.

**Results:**

TgASP1 appears to be a membrane protein located primarily at the tip of the *T. gondii* tachyzoite. Investigation of its potential as a DNA vaccine showed that it elicited strong humoral and cellular immune responses in mice, and that these responses were mediated by Th-1 cells. Mice immunized with the vaccine had greater levels of protection against mortality following challenge with *T. gondii* RH tachyzoites than did those immunized with PBS or the empty vector control.

**Conclusions:**

TgASP1 is a novel candidate DNA vaccine that merits further investigation.

## Background

*Toxoplasma gondii*, a coccidian apicomplexan, is an obligate intracellular parasite of humans and other warm-blooded animals [1,2]. *T. gondii* infection normally causes mild symptoms or is asymptomatic in humans, but toxoplasma encephalitis has emerged as one of the most frequent opportunistic infections in HIV-infected patients [3]. In addition, *T. gondii* infection during pregnancy carries a high risk of congenital toxoplasmosis in infants or acute retinochoroiditis in pregnant women [4].

*T. gondii* expresses several proteases, including cysteine proteases, aspartic proteases and serine proteases. The parasite has five cysteine proteases, including one cathepsin B-like (TgCPB), one cathepsin L-like (TgCPL) and three cathepsin C-like (TgCPC1, 2 and 3) proteins. *T. gondii* also has four aspartic protease encoding genes that are designated toxomepsin 1, 2, 3 and 5 (TgASP1, 2, 3 and 5, respectively) and two subtilases (TgSUB1 and TgSUB2). *T. gondii* also possesses five genes encoding a family of polytopic membrane rhomboid-like serine proteases, which are known as TgROM 1 to 5 [5].

Proteases play several key roles during *T. gondii* infection, including host cell invasion, nutrient acquisition, avoidance of host protective immune responses, escape from the parasitophorous vacuole (PV), parasite differentiation, and regulation of pathogenesis [6-9]. As an obligatory intracellular protozoan parasite, host cell invasion is a prerequisite for establishing and maintaining a life-long infection in its host. Several lines of evidence suggest that proteases are important during invasion by apicomplexan parasites, and proteases are considered critical for assembly and trafficking of organellar-related proteins [5,10]. Proteases targeted to the *Plasmodium* food vacuole, a unique organelle dedicated to hemoglobin degradation, are critical to parasite survival in these apicomplexan parasites [11]. Cysteine proteases can modulate host immune responses by altering the normal function of key receptors or pathways in the mammalian immune system [12]. In addition, a variety of data have been published suggesting that proteases are involved in many other activities, including for example, antigen presentation [13].

Aspartic proteases are common in eukaryotes where they are known to play key roles in a wide range of biological functions. There are four aspartic protease genes in the *T. gondii* genome that are expressed in tachyzoites. TgASP3 and TgASP5 are localized to the Golgi compartment, but TgASP1 is only found in *T. gondii* and the related apicomplexan, *Neospora caninum*[14]. TgASP1 is synthesized as a zymogen [15], and its role in the food vacuole is related to hemoglobin degradation. TgASP1 re-localizes to the nascent inner membrane complex (IMC) of daughter cells before coalescing at the end of parasite cell division [16].

Toxoplasmosis, caused by *T. gondii*, is a serious threat to human health; however, there are no drug treatments available that can cure it. Hence, other options for controlling the disease are sought. DNA vaccines are an option worth considering as they can induce continuous and strong protective immune responses against this ubiquitous parasite [17,18].

In this study, we constructed a TgASP1 gene vaccine to evaluate protective immune responses against toxoplasmosis in laboratory mice. Here, we show that TgASP1 is a novel vaccine candidate that can induce substantial humoral and cellular immune responses against *T. gondii* infections in mice.

## Methods

### Bioinformatics analysis of TgASP1

TgASP1 nucleotide (GenBank ID: AY580011.1) and amino acid sequences (GenBank ID: AAS90335.1) were obtained from GenBank (http://www.ncbi.nlm.nih.gov/genbank) and were analyzed using DNAMAN software and BLAST (protein-protein). GENSCAN was used to search for the open reading frame (ORF) structure of the TgASP1 gene (http://genes.mit.edu/GENSCAN.html), whilst the physical and chemical properties of the protein were analyzed by ProtParam (http://web.expasy.org/protparam/). The transmembrane structure and presence of signal peptides were predicted by the TMHMM server (http://www.cbs.dtu.dk/services/TMHMM-2.0/) and SignalP Server (http://www.cbs.dtu.dk/services/SignalP/), respectively. B-cell epitopes were predicted using DNASTAR, Gene Runner and DNAMAN software, while the 2D and 3D structures were determined by SOPMA (http://npsa-pbil.ibcp.fr/cgi-bin/npsa_automat.pl?page=/NPSA/npsa_sopma.html) and I-TASSER (http://zhanglab.ccmb.med.umich.edu/I-TASSER/).

### Parasites and mice

The *T. gondii* RH strain was maintained and passaged *in vitro* in human malignant epithelial cells (HeLa cells), which were cultured in a mixture of Dulbecco’s Modification of Eagle’s Medium (DMEM, Gibco) and nutrient supplement containing 10% heat-inactivated fetal bovine serum (FBS, HyClone), 2 mM L-glutamine, penicillin (100 U/ml) and streptomycin (100 μg/ml). Cell cultures were maintained at 37°C in a 5% CO_2_ environment and were changed every 2 to 3 d. The *T. gondii* RH strain was harvested and purified as previously described [19,20]. Briefly, tachyzoites were collected by washing in cold phosphate buffered saline (PBS), centrifuged, resuspended in cold PBS and syringed three times with a 27-gauge needle. The parasites were filtered through a 5.0 μm pore size filter (Millipore, USA), washed with cold PBS and pelleted at 1500 rpm for 10 min. Genomic DNA was extracted from tachyzoites prepared by this method.

Six-week-old female BALB/c mice were purchased from the Shandong University Laboratory Animal Center. All mice were maintained under specific-pathogen-free (SPF) conditions when the first immunizations were conducted. All the animal experiments were approved by the Animal Ethics Committee of Shandong University.

### Expression plasmid construction

The TgASP1 gene was amplified from *T. gondii* genomic DNA by polymerase chain reaction (PCR) using the following primer pairs. TgASP1 for construction of the prokaryotic expression plasmid: 5′-cggGGTACCATGTCT-CCGTCGTCGCG-3′ (forward) and 5′-cccAAGCTTTCAGTTCTTGAGTCTG GCGA-3′ (reverse), which contains *Kpn*I and *Hin*dIII restriction sites (underlined), and TgASP1 for construction of the eukaryotic expression plasmid: 5′-ccgCTCGAGATGTCTCCGTCGTCGCG-3′ (forward), 5′- cggGGTACCTCAGTTCTTGAGTCTGGCGA-3′ (reverse), which contains *Xho*I and *Kpn*I restriction sites (underlined). PCR products were cloned into the pEASY-T1 simple vector (TransGen Biotech, China) to generate a recombinant cloning plasmid. After sequencing, TgASP1 was subcloned into the prokaryotic expression plasmid pET-30a(+) (Novagen, USA), and into the eukaryotic expression plasmid pEGFP-C1 (Clontech, USA) to produce pET-30a-TgASP1 and pEGFP-TgASP1 (pTgASP1), respectively.

### Preparation of anti-rTgASP1 sera

The recombinant plasmid pET-30a-TgASP1 was transformed into *E.coli* BL21 (DE3) cells and grown in Luria Bertani medium (LB) with kanamycin (25 μg/ml). Recombinant TgASP1 (rTgASP1) protein was induced with 1 mM isopropyl-β-D-thiogalactoside (IPTG) for 6 h at 25°C. The cells were lysed with 50 mM Tris pH7.4, 150 mM NaCl, 1% Triton X-100, 2mM EDTA containing 1 mM of the protease inhibitor PMSF (phenylmethanesulfonyl fluoride) and then centrifuged at 4°C at 10,000 × *g* for 15 min, after which the protein was purified by binding of its carboxy terminal histidine (His) tag to Ni-NTA resin (Sangon Biotech, China).

BALB/c mice were immunized subcutaneously with 100 μg of purified rTgASP1 in an equal volume of Freund’s complete adjuvant (Sigma) for the first injection. The second and third injections consisted of 50 μg of purified protein in Freund’s incomplete adjuvant (Sigma). Antisera were collected from the mice two weeks after their last immunizations. Immunoglobulin G (IgG) was purified form the antisera using protein A chromatography columns (GE Healthcare, USA), and the IgG containing fractions were identified by SDS-PAGE and western blotting as previously described [21]. Approximately 500 ng of rTgASP1 protein was used in sodium dodecylsulfate-polyacrylamide gel electrophoresis (SDS-PAGE). After electrophoresis, the separated protein bands were transferred onto polyvinylidene difluoride (PVDF) membranes (Millipore, USA), which were blocked with 5% (W/V) skimmed milk diluted in PBS for 2 h, after which they were incubated with a mouse anti-TgASP1 antibody (dilution 1:500) for 24 h at 4°C. After washing in PBS-T (PBS pH 7.4 containing 0.05% Tween 20), the membrane was incubated with diluted goat anti-mouse IgG horseradish peroxidase (HRP)-labeled secondary antibody (1:10,000; Sigma, USA) for 1 h. Protein bands were detected with ECL chemiluminescence reagents (Cowin Biotech, China).

### Immunolocalization experiments

Indirect IFAs were performed on intracellular and extracellular parasites as described previously [22,23]. Briefly, each parasite type was grown overnight in HeLa cells and then fixed on slides with 4% paraformaldehyde for 20 min, washed with PBS, permeabilized with 0.2% Triton X-100 in PBS for 20 min, and then blocked in 10% FBS for 20 min. After washing, the cells and parasites were stained with the primary antibody (anti-rTgASP1, 1:500, diluted in PBS-FBS) for 1 h, washed with PBS, and then incubated with the secondary antibody (Alexa Fluor 647-Labeled Goat anti-mouse IgG, Beyotime, China) diluted in 1% PBS-FBS (dilution 1:1000) for 1 h. After washing with PBS, the slides were mounted in Antifade Mounting Medium (Beyotime, China) and observed under a laser scanning confocal microscope (Carl Zeiss LSM780, Germany).

### TgASP1 expression in mammalian cells

HEK293 cells were transfected with pEGFP-TgASP1 using Lipofectamine 2000 reagent (Invitrogen, USA) as previously described [24]. Before transfection, HEK293 cells were transferred into 6-well culture plates (Costar, USA) and cultured until the density of the cells reached approximately 80%. Liposomes (10 μl) were diluted in 240 μl of DMEM and then incubated at room temperature for 5 min. The solution was gently mixed with 4 μg of plasmid DNA in 250 μl of DMEM and then incubated at room temperature for 20 min. DNA-lipid complexes were added to the cells that had been rinsed in serum-free medium. After incubation for 6 h at 37°C in 5% CO_2_, the medium was exchanged with that containing 10% FBS. After 48 h, the transfected HEK293 cells were collected and observed under a fluorescence microscope (Carl Zeiss). The cells were lysed with RIPA buffer (50 mM Tris pH 7.4, 150 mM NaCl, 1% Triton X-100, 1% Sodium deoxycholate, 0.1% SDS) containing 1 mM of PMSF and centrifuged at 12,000 × *g* for 10 min, at 48 h after transfection. The products were visualized by SDS-PAGE and western blotting. Approximately 500 ng of purified rTgASP1 protein was separated by SDS-PAGE. The separated protein bands were transferred onto PVDF membranes. The procedures used were the same as those described in the section “Preparation of anti-rTgASP1 sera”.

### Experimental design

Thirty-nine mice were randomly divided into three groups (13 per group) as described previously [25]. Mice were injected intramuscularly in their hind legs four times, at 2 weekly intervals. Group 1 (n = 13), were immunized with 100 μg of the pEGFP-TgASP1 plasmid DNA resuspended in 100 μl of sterile PBS. Group 2 (n = 14) were immunized with 100 μg of the empty vector resuspended in 100 μl of sterile PBS (control group), whereas group 3, which also served as a control group (n = 13), received 100 μl of sterile PBS alone. Two weeks after the final immunizations, 3 mice per group were euthanized and their spleens were isolated. The remaining mice were challenged intraperitoneally with 100 μl phosphate-buffered saline (PBS) containing 1 × 10^4^*T. gondii* tachyzoites and the survival time and condition of each mouse was recorded.

### Serum samples and antibody assays

Serum samples were collected from all of the mice prior to each immunization. The serum was tested for total IgG, IgG1 and IgG2a. IgG antibody levels against *T. gondii* were measured by enzyme-linked immunosorbent assay (ELISA) [26,27]. In brief, microtiter plates (Costar, USA) were coated overnight with rTgASP1 protein (10 pmol/well) in 50 mM carbonate buffer (pH 9.6). Plates were washed with PBS-T three times and were blocked with 1% Bovine Serum Albumin (BSA) for 1 h at 37°C. After three washes, the plates were incubated with mouse sera diluted in PBS for 1h at 37°C. After three washes, goat anti-mouse IgG, IgG1 or IgG2a secondary antibodies conjugated with horseradish peroxidase (Sigma, USA) were added and the plates were incubated at 37°C for 1 h. Immune complexes were observed by incubation with orthophenylene-diamine (Sigma, USA) and 0.15% H_2_O_2_ for 30 min. Reactions were stopped by adding 2M H_2_SO_4_ and the results were read with an ELISA reader (Bio-tekEL × 800, USA) set at 490 nm. The results were expressed as the optical density (OD) ratio between the OD of a sample divided by the OD of the antibody-free control. All samples were run in triplicate.

### Cytokine assays

Cytokine levels were determined according to the method described previously [28]. Spleens were isolated from three mice per group 2 weeks after their last immunizations. A single-cell suspension was prepared by crushing the spleens through stainless steel meshes followed by suspension in lysis buffer (0.83% NH_4_Cl, 0.01 M Tris–HCl, pH7.2). The cells were then suspended in RPMI 1640 medium (Sigma, USA) supplemented with 10% FBS, 100 U/ml of penicillin and 100 μg/ml of streptomycin. Cell densities were adjusted to 1 × 10^6^ cells/ml, after which they were dispensed into 96-well plates at 37°C in 5% CO_2_, and the cell-free supernatants were harvested and assayed for interleukin-4 (IL-4) at 24 h, interleukin-10 (IL-10) at 72 h, and gamma interferon (IFN-γ) at 96 h using an ELISA kit (R&D Systems, USA).

### Challenge infections

Two weeks after their last immunizations, the mice were challenged intraperitoneally with 1 × 10^4^*T. gondii* tachyzoites and the survival rate and condition of the mice was monitored daily. Toxoplasmosis signs, i.e., food and water intake difficulties, lethargy and severe ascites, were used to determine when the animals should be humanely euthanized. Mice that showed signs of illness were euthanized by containment in a chamber where a CO_2_ concentration of 60% to 70% was administered over a 5-min exposure time [29]; this was followed by cervical dislocation if required.

### Statistical analysis

Statistical analysis in all of the groups was performed using SPSS software. The levels of antibody and cytokine production among the different groups were analyzed and determined by a one-way ANOVA. Survival time for the mice was compared using the Kaplan-Meier method. The difference was considered statistically significant if the P value was less than 0.05.

## Results

### Bioinformatics analysis

**Figure 1:**
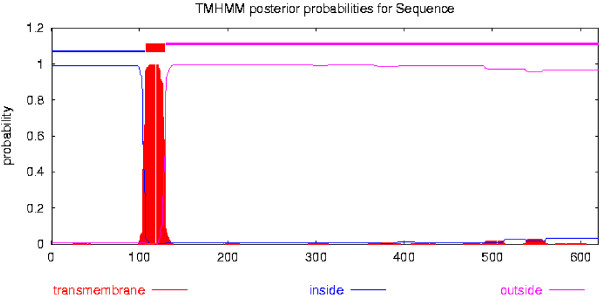
**Alignment of ASP1 protein sequences from toxoplasma strains.** Red letters indicate amino acid differences, a blue letter shows a missing amino acid, while * represents identical amino acids.

**Figure 2:**
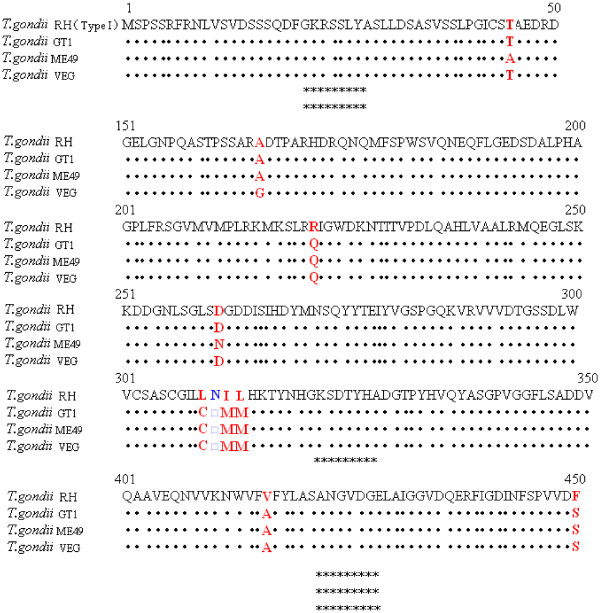
**Prediction of transmembrane helices in TgASP1.** The analysis indicates that TgASP1 has a transmembrane structure.

**Figure 3:**
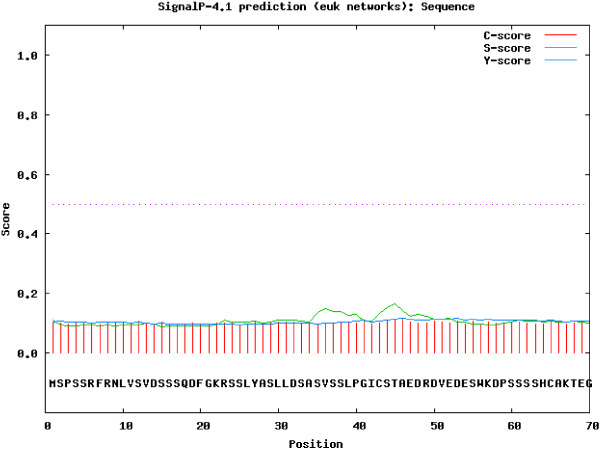
**Prediction of the signal peptide cleavage site locations in the TgASP1 protein.** The analysis indicates that TgASP1 does not contain a signal peptide.

TgASP1 comprises a 620 amino acid protein with a molecular weight of 67.1367 kDa. The theoretical isoelectric point and instability index were calculated at 6.34, and 42.98, respectively, while the aliphatic index and the grand average of hydropathicity (GRAVY) were 84.29% and −0.072, respectively. A protein sequence alignment of TgASP1 for four *T. gondii* strains is shown in Figure 1. The alignment shows that the ASP1 proteins from these strains share 98.3% similarity. ASP1 of the *T. gondii* RH strain shares 98.8% sequence identity with *T. gondii* GT1, 98.5% with *T. gondii* ME49 and 98.7% with *T. gondii* VEG. Transmembrane domain and signal peptide cleavage site predictions for TgASP1 are shown in Figures 2 and 3, respectively.

### Secondary structure and linear-B cell epitope prediction

Epitopes are the foundations of protein antigenicity that determine antigen specificity. Many types of epitope prediction methods can provide clues about the hydrophilicity, antibody accessibility, antigenicity, flexibility, charge distribution and secondary structure of a protein. Despite the lack of an infallible method to predict antigenic epitopes, several rules can be followed to establish which peptide fragments of a protein are likely to be antigenic. First, antigenic epitopes should be located in solvent-accessible regions and contain both hydrophobic and hydrophilic residues. Second, peptides lying in long loops connecting secondary structure motifs are preferable, while peptides located in helical regions should be avoided. Whenever possible, peptides that are in the N- and C-terminal regions of a protein should be chosen because they are usually solvent accessible and unstructured.

**Figure 4:**
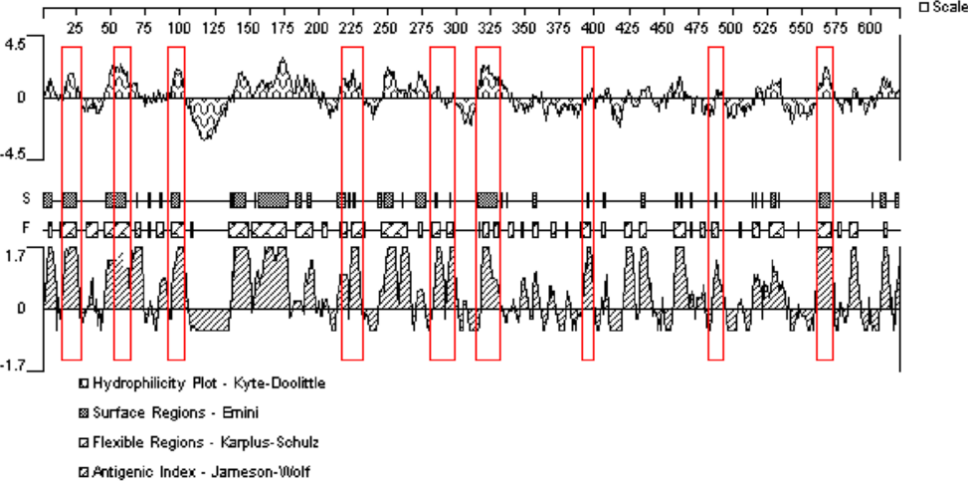
Prediction of B-cell epitopes in the TgASP1 protein based on DNAStar analysis.

Following the guidelines above, we analyzed TgASP1 to identify liner-B cell epitopes using DNASTAR software and chose peptides that had good hydrophilicity, high accessibility, satisfactory flexibility and strong antigenicity (Figure 4). The results showed the presence of several peptides that had potential to be suitable epitopes in TgASP1. To verify these results, we used DNAMAN software to analyze the sequences and we selected nine potential epitopes with the highest antigen index scores. The details of this analysis are shown in Table 1.

**Table 1:** Analysis of linear-B cell antigenic epitopes on TgASP1

**Order**	**Position**	**Sequences**	**Score**
1	13-26	VDSSSQDFGKRSSL	1.140
2	61-67	SSSHCAK	1.090
3	98-105	RRSLGKAV	1.211
4	222-231	IGWDKNTITV	1.169
5	276-294	YTEIYVGSPGQKVRVVVDT	1.193
6	316-331	TYNHGKSDTYHADGTP	1.136
7	398-407	PFMQAAVEQN	1.203
8	486-492	LDEVKRI	1.141
9	561-571	EGGRPTPQKNG	1.126

### Modeling the three-dimensional (3-D) structure of TgASP1

**Figure 5:**
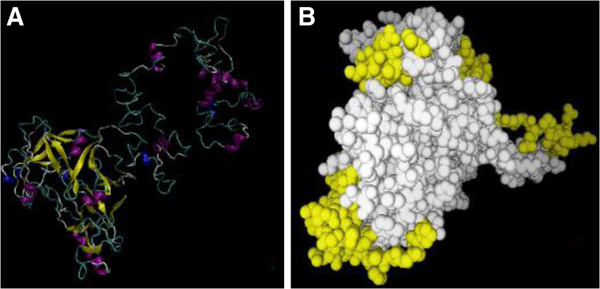
**3D structure predictions for TgASP1.** (**A**) 3-D structure of TgASP1. The 3D model with the highest score for the TgASP1 protein was selected. The model was viewed by VMD software and colored to illustrate the secondary structure components (yellow: β-strands, purple: α-helix, gray: coil); (**B**) The distribution of potential epitopes on TgASP1 is marked with yellow balls.

Modeling the potentially antigenic regions of a protein as a 3-D structure increases the accuracy of epitope prediction, and assists with determining the epitope boundaries. This process also aids identification of predicted conformational epitopes. We used SOPMA and I-TASSER on-line services to predict the 2-D and 3-D structures of TgASP1 (Figure 5). The results predicted that TgASP1 contains 14.6% alpha helix, 33.33% extended strand, 9.73% beta turn and 42.58% random coil. These unconsolidated regions that are composed of many β-turns and random coils have potential for forming antigenic epitopes.

### rTgASP1 antibody specificity

**Figure 6:**
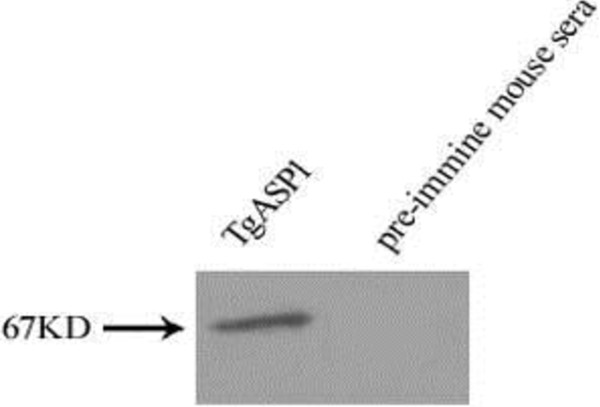
Western blot of native TgASP1.

Western-blotted PVDF membranes were incubated separately with mouse anti-TgASP1 antibody or pre-immune sera. The results indicate that the mouse anti-TgASP1 antibody recognized a protein of around 67 kDa, which is consistent with the predicted size of the TgASP1 protein (Figure 6).

### Localization of native TgASP1 protein in *T. gondii* tachyzoites

**Figure 7:**
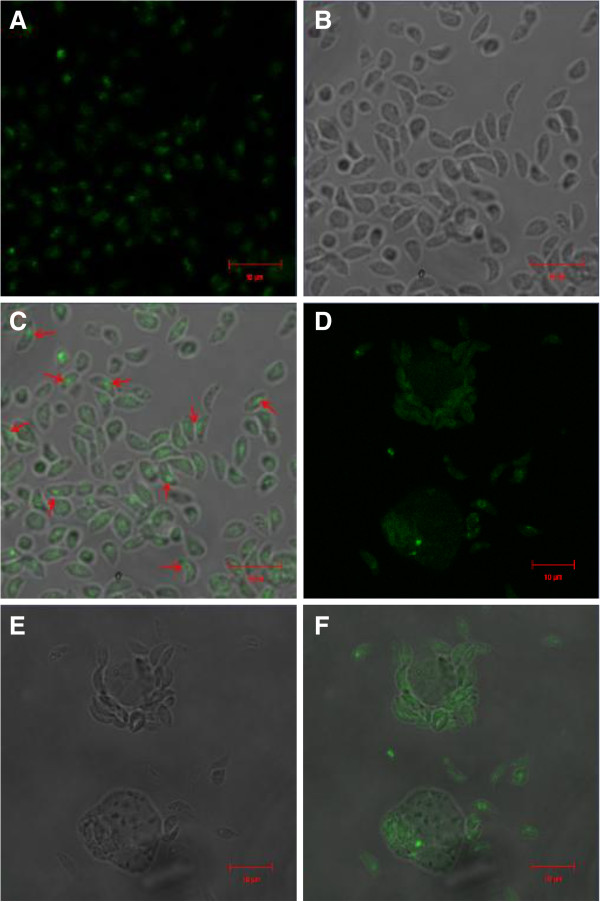
**Immunofluorescence staining of intracellular and extracellular parasites.** Extracellular parasites were observed under a light microscope (**A**), fluorescence microscope (**B**), and using merged channel fluorescence (**C**). Intracellular parasites were observed under a light microscope (**D**), fluorescence microscope (**E**) and using merged channel fluorescence (**F**).

Extracellular and intracellular parasites were analyzed by IFAs. Extracellular parasites were blotted with the mouse anti-rTgASP1 antibody and observed under a light microscope (Figure 7A), a fluorescence microscope (Figure 7B) and a merged channel (Figure 7C). Intracellular parasites were analyzed by the same method (Figure 7D,E and F). The results show that TgASP1 is located mainly at the tip of the tachyzoite, as opposed to being widely distributed in the cytoplasm or secreted from the parasite.

### Identification of pTgASP1 expression products

**Figure 8:**
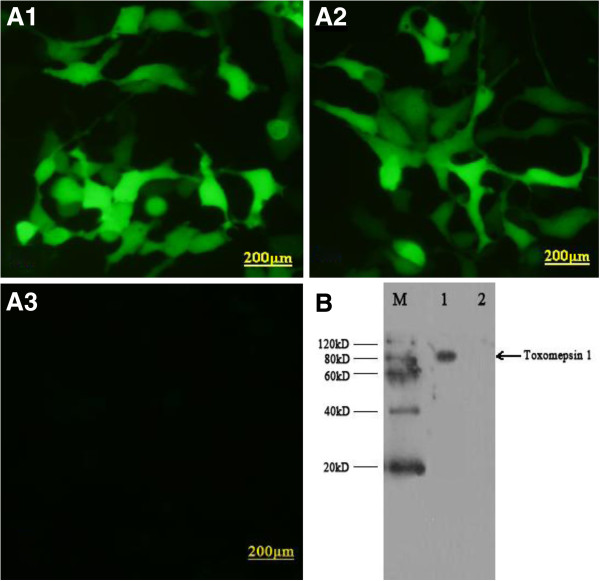
**Expression of TgASP1 in HEK293 cells as detected by indirect fluorescent antibody testing and western blotting.** (**A1**) pTgASP1-transfected HEK293 cells; (**A2**) empty pEGFP vector-transfected HEK293 cells; (**A3**) non-transfected HEK293 cells; (**B**) M: protein marker; lane 1 HEK293 cells transfected with pTgASP1; lane 2 HEK293 cells transfected with empty pEGFP vector.

*In vitro* expression of pTgASP1 was evaluated by IFA at 48h post-transfection. As shown in Figure 8 (A1 and A2), green fluorescence was observed in HEK293 cells transfected with pEGFP-TgASP1 or pEGFP, whereas no fluorescence was observed in the non-transfected HEK293 cells (A3). Western blotting revealed that the rTgASP1 protein (~67 kDa in size including the 27 kDa green fluorescent protein) was detected in HEK293 cells transfected with pTgASP1 (B), but not in cells transfected with the empty pEGFP vector.

### Antibody responses in immunized mice

**Figure 9:**
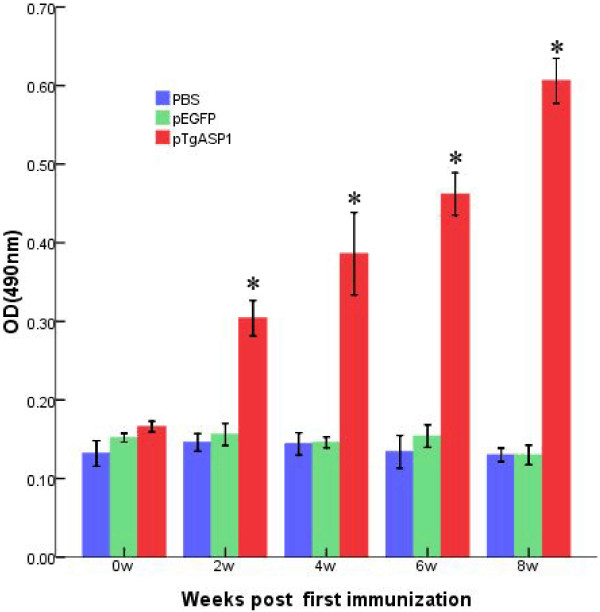
**IgG antibody responses in pTgASP1-immunized mice.** Sera were collected 1 d prior to each immunization and screened by ELISA. Results are shown as the means of the OD 490 ± SD and statistical differences (P < 0.05) are indicated by * as compared with PBS or pEGFP.

As shown in Figure 9, statistically significant high levels of IgG antibodies were observed in the pTgASP1-vaccinated group, the levels of which gradually increased with each successive immunization. The levels were higher than those of the control groups that had been immunized with PBS or pEGFP. A statistically significant difference was found between the experimental group and the control groups (P < 0.05). The result indicates that the recombinant plasmid encoding TgASP1 induced a strong IgG antibody response in the vaccinated mice, with the OD values reaching high levels two weeks after the third immunization.

**Figure 10:**
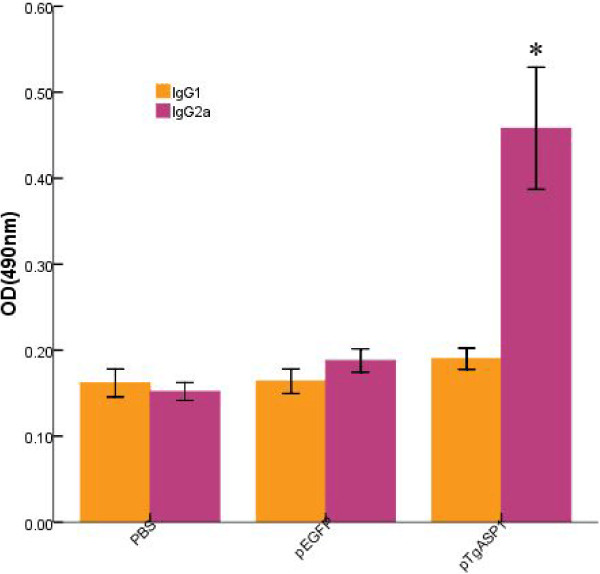
**Distribution of IgG1 and IgG2a subtypes in pTgASP1-immunized mice.** Serum levels of IgG1 and IgG2a were analyzed by ELISA two weeks after the final immunizations. Results are expressed as the means of the OD 490 ± SD and statistically significant differences (P < 0.05) are indicated by an asterisk (*), as compared with control groups.

The IgG1 and IgG2a subgroup antibody levels for all of the groups in the second week after the final immunizations are shown in Figure 10. IgG1 levels were not significantly different between the TgASP1-immunized and control groups (PBS or pEGFP) (P > 0.05); however, mice immunized with pTgASP1 generated higher levels of IgG2a than those immunized with PBS or pEGFP (P < 0.05).

### Cytokine production

Splenocyte supernatants harvested at different time points were used to measure cytokine levels (IFN-γ, IL-4 and IL-10) in the different groups. As shown in Table 2, mice vaccinated with pTgASP1 generated significantly higher IFN-γ levels than mice vaccinated with PBS or the empty vector (P < 0.05). In contrast, similarly low levels of IL-4 and IL-10 were measured in the groups and no statistically significant differences were found among them (P > 0.05).

**Table 2:** **Cytokine production in cultures of splenocytes**^**a **^**from immunized BALB/c mice**

**Group**	**Cytokine production (pg/ml)**^**b**^		
	**IFN-γ**	**IL-4**	**IL-10**
PBS	48.03 ± 1.86	37.31 ± 1.76	40.97 ± 1.03
pEGFP	44.32 ± 4.43	36.58 ± 2.42	41.60 ± 2.01
PTgASP1	715.76 ± 23.37*	37.36 ± 1.09	38.53 ± 0.93

^a^Splenocytes from 3 mice per group on the 4th week after the final immunization.

^b^Values for IFN-γ are for 96 h. Values for IL-10 are for 72 h. Values for IL-4 are for 24 h.

*Compared with PBS-adjuvant or pEGFP controls, P < 0.05.

### Protective efficacy of DNA vaccination against *T. gondii* in mice

**Figure 11:**
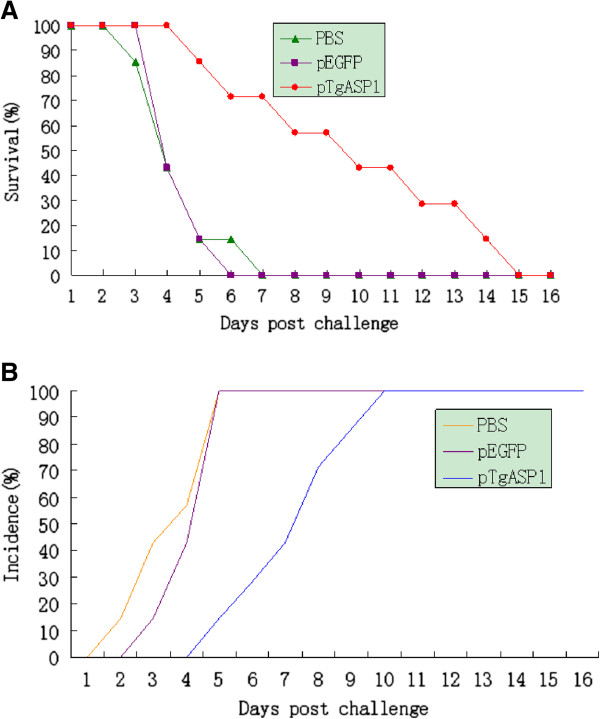
**Survival curve for pTgASP1-immunized BALB/c mice following challenge with *T. gondii*.** (**A**) Three groups of mice were challenged with 1 × 10^4^ tachyzoites of the virulent RH strain of *T. gondii* two weeks after their last immunization. n = 10 per group. Survival was monitored daily for 16 d after challenge. (**B**) The incidence of infection in the three groups of mice.

All mice were challenged intraperitoneally with the *T. gondii* RH strain to evaluate the degree of immunoprotection induced by the DNA vaccine. Mice were checked daily until all of them required euthanasia. As shown in Figure 11A, immunization with the DNA vaccine dramatically increased the survival time of the vaccinated group in comparison to the control groups that were vaccinated with PBS or pEGFP (P < 0.05). In addition, as shown in Figure 11B, the infection onset times in the immunized mice were later than mice in the control groups.

## Discussion

Bioinformatics can play a central role in the analysis and interpretation of genomic and proteomic data. It uses methods and technologies from mathematics, statistics, computer sciences, physics, biology and medicine [30]. It can be a powerful tool for predicting the structure and function of a protein from its amino acid sequence by means of its similarity to a sequence of known structure or function. This method plays a major role in guiding the experimental characterization of a genome [31], and, because of its effectiveness and low cost, bioinformatics has been widely used to predict antigenic epitopes on proteins [32].

Bioinformatics analysis has allowed us to predict that TgASP1 is a soluble transmembrane protein that is conserved between different strains of *T. gondii*. The prediction results suggest that TgASP1 has no signal peptide sequence, which should improve the secretion efficiency of anti-rTgASP1 antibodies [33]. We used several different software packages and on-line services to predict the protein sequence and 3D structure of TgASP1, and identified several promising linear-B cell epitopes and potential Th-cell epitopes. The data obtained indicate that TgASP1 could hold promise as a protective antigen.

The result of the indirect immunolocalization experiment suggests that TgASP1 is apically located in the parasite, while the bioinformatics analysis suggests that it is a membrane protein. However, TgASP1 does not appear to localize to regions in the parasite where the secretory organs are located, such as the rhoptries, micronemes and dense granules. Previous studies have suggested that TgASP1 resides in a novel compartment of the secretory system that potentially serves as a link between the Golgi and the IMC [16].

The results of the immunization experiments show that the pTgASP1 plasmid can induce strong humoral and cellular immune responses. Importantly, significantly higher levels of total IgG antibodies were observed in the pTgASP1-immunized group than were observed in the control groups immunized with PBS or the pEGFP vector. In addition, pTgASP1 induces high levels of IgG2a and IFN-γ, but low levels of IgG1, IL-4, and IL-10. Th cells can be divided into two subpopulations known as Th-1 and Th-2 cells. Th-1 cells help activate cytotoxic T cells (Tc cells), whereas Th-2 cells perform as B cell helpers. IFN-γ production favors Th1-type immune responses and Th1 cells secrete IFN-γ and IL-2. IL-4 favors Th2 responses and Th2 cells secrete IL-4 and IL-10 [34,35]. Therefore, our results indicate that the cellular immune response induced by the pTgASP1 single-gene vaccine may be directed towards a Th1-type response in BALB/c mice. However, there are many factors that can influence the direction of differentiation for Th cells. For example, IL-12 and IL-18 can also favor Th1-type immune responses and Th-2 cells can also enhance the levels of IL-5, IL-6, IL-9 and IL-13 [36]. These factors were not investigated in this study, however.

We recorded the survival time of all the mice in the three groups after intraperitoneal challenge with 1 × 10^4^ tachyzoites of the virulent RH strain of *T. gondii*. Compared with the control groups, the pTgASP1-immunized mice showed greater protection from *T. gondii* infection and longer survival rates than the control groups, which needed to be euthanized within 8 days of challenge with the parasites. However, the surviving mice needed to be euthanised by the 15^th^ day post-challenge, indicating that the DNA vaccine did not provide complete protection. Nevertheless, DNA vaccines should be explored further as a strategy for the control of *T. gondii* infection.

Current research on vaccine antigens has focused mainly on *T. gondii-*specific component antigens, which include the surface antigen (SAG) [37], granule protein (GRA) [38], rhoptry protein (ROP) [39], micronemal protein (MIC) [40], apical membrane antigen (AMA) [41], and rhomboid-like protease (ROM) [42]. Attention has also been focused on obtaining epitope-based vaccines by means of cDNA library screening. In addition, some viruses or bacteria have been used as carriers for live vector vaccines. One example is the recombinant MVA/ROP2 vaccinia virus, which carries the *T. gondii* ROP2 gene that has been shown to induce strong immune protection against *T. gondii* in mice [43]. Another example is the GRA1 antigen that was used with the *Mycobacterium bovis* Bacillus Calmette Guerin (BCG) to build a recombinant BCG-GRA1 vaccine against *T. gondii* infection [44]. While looking for potentially useful candidate antigens, researchers have also tried to improve the level of immune protection against *T. gondii* disease through the use of vaccine adjuvants. Such adjuvants include Freund’s, cholera toxin, IL-12 [45], hyaluronidase (HAase) [46] and CpG-oligodeoxynucleotides [47].

## Conclusions

The starting point for this study was using bioinformatics to analyze the TgASP1 protein to identify potential antigenic epitopes. Several promising epitopes were identified using this approach, indicating that in silico approaches can be useful for epitope prediction. Thereafter, we constructed a pTgASP1 single-gene vaccine to evaluate the level of immunoprotection induced by it in mice. The results of this study show that mice vaccinated with the pTgASP1 DNA vaccine survived longer after infection with tachyzoites of the virulent RH strain of *T. gondii* than did the controls. However, the vaccine did not afford complete protection. Nevertheless, DNA vaccines require further investigation as a strategy for controlling *T. gondii* infection.

## Competing interests

The authors declare that they have no competing interests.

## Authors’ contributions

GZ carried out the experiments and drafted the manuscript. GL and MM contributed to the revised version of the manuscript. YB, MS, LW, YH, QZ, HC, XQZ and HZ helped carry out various aspects of the experiments and revised the manuscript. SH and AZ conceived and designed the study and made critical revisions to the manuscript. All authors have read and approved the final manuscript.
